# Deformed Epidermal Autoregulatory Factor-1 (DEAF1) Interacts with the Ku70 Subunit of the DNA-Dependent Protein Kinase Complex

**DOI:** 10.1371/journal.pone.0033404

**Published:** 2012-03-19

**Authors:** Philip J. Jensik, Jodi I. Huggenvik, Michael W. Collard

**Affiliations:** Department of Physiology, Southern Illinois University School of Medicine, Carbondale, Illinois, United States of America; Roswell Park Cancer Institute, United States of America

## Abstract

Deformed Epidermal Autoregulatory Factor 1 (DEAF1) is a transcription factor linked to suicide, cancer, autoimmune disorders and neural tube defects. To better understand the role of DEAF1 in protein interaction networks, a GST-DEAF1 fusion protein was used to isolate interacting proteins in mammalian cell lysates, and the XRCC6 (Ku70) and the XRCC5 (Ku80) subunits of DNA dependent protein kinase (DNA-PK) complex were identified by mass spectrometry, and the DNA-PK catalytic subunit was identified by immunoblotting. Interaction of DEAF1 with Ku70 and Ku80 was confirmed to occur within cells by co-immunoprecipitation of epitope-tagged proteins, and was mediated through interaction with the Ku70 subunit. Using *in vitro* GST-pulldowns, interaction between DEAF1 and the Ku70 subunit was mapped to the DEAF1 DNA binding domain and the C-terminal Bax-binding region of Ku70. In transfected cells, DEAF1 and Ku70 colocalized to the nucleus, but Ku70 could not relocalize a mutant cytoplasmic form of DEAF1 to the nucleus. Using an *in vitro* kinase assay, DEAF1 was phosphorylated by DNA-PK in a DNA-independent manner. Electrophoretic mobility shift assays showed that DEAF1 or Ku70/Ku80 did not interfere with the DNA binding of each other, but DNA containing DEAF1 binding sites inhibited the DEAF1-Ku70 interaction. The data demonstrates that DEAF1 can interact with the DNA-PK complex through interactions of its DNA binding domain with the carboxy-terminal region of Ku70 that contains the Bax binding domain, and that DEAF1 is a potential substrate for DNA-PK.

## Introduction

Deformed Epidermal Autoregulatory Factor 1 (DEAF1) is a transcription factor linked to suicide [1], [2], [3], cancer [4], [5], autoimmune disorders [6] and neural tube defects [7]. DEAF1 was first identified in *Drosophila* as a DNA binding protein that recognizes direct repeats of TTCG within the transcriptional promoter of the hox gene *Deformed* [8]. DEAF1 binds to DNA with TTCG motifs through an alpha-helical, surface patch containing the positively charged amino acid sequence KDWK that is located within a conserved structural fold called the “SAND” domain [9]. In addition to the SAND domain, the DEAF1 DNA binding domain also consists of a nuclear localization signal (NLS) and a zinc binding motif which function in DEAF1-DEAF1 protein interaction [10]. A second DEAF1-DEAF1 interaction domain occurs in a region adjacent to the DNA binding domain and contains a leptomycin B sensitive nuclear export signal (NES) [10]. The NES, the NLS and DEAF1-DEAF1 protein interaction provide a mechanism for shuttling the protein between the cytoplasmic and nuclear compartments where DEAF1 may have distinct functions. Protein-protein interactions are also likely to affect location and function. The LIM only protein 4 (LMO4) was shown to interact with amino acids 334–588 of mouse Deaf1 [11]. This region of DEAF1 includes the nuclear export signal and the second DEAF1-DEAF1 interaction domain. LMO4 interactions were also observed with the N-terminal domain of DEAF1, although they appeared to be weaker. Using yeast two-hybrid experiments and GST pulldown analysis, DEAF1 was also shown to interact with LIM domain binding protein 1 (LDB1/CLIM2) [11].

In this study, we use GST-DEAF1 pulldowns from cell lysates followed by mass spectrometric analysis to isolate and identify DEAF1 protein interaction with the Ku70 subunit of the DNA dependent protein kinase (DNA-PK) complex. DNA-PK is a heterotrimeric protein complex composed of three subunits: Ku70, Ku80, and the DNA-PK catalytic subunit (DNA-PKcs). The protein regions necessary for DEAF1 and Ku70 interaction were identified. We also examined the ability of DNA-PK to phosphorylate DEAF1 and the effects of DNA on DEAF1 and Ku70 protein-interactions.

## Materials and Methods

### Reagents and Antibodies

Glutathione (GSH)-Sepharose beads were purchased from (GE Healthcare). Cell lines were purchased from American Type Culture Collection (ATCC): monkey kidney CV1 (CCL-70); human embryonic kidney 293T/17 (CRL11268); and human prostate PC-3 (CRL1435). Antibodies to the epitopes FLAG and HA, anti-FLAG coupled beads, and FLAG peptide were purchased from Sigma-Aldridge and antibodies to DNA-PKcs were purchased from Lab Vision. Antibodies to full-length human DEAF1 were produced in rabbits and has been as previously described [12].

### Plasmids

DEAF1 constructs were derived from human DEAF1 cDNA, accession number AF049459 and have been described (DEAF1 in pcDNA3, GST-DEAF1, and K304T [12]; DEAF1-FLAG [10]). Full-length cDNA clones for Ku70 (XRCC6, MGC-15860) and Ku80 (XRCC5, ATCC # 7516094) were obtained from ATCC. DNA fragments, both full-length and deletions of DEAF1, Ku70, and Ku80 were generated by PCR and subcloned into plasmids to produce in-frame fusions with GST, GFP, HA, and FLAG. All plasmid constructs were verified by DNA sequencing.

### Purification of GST-tagged and FLAG-tagged Proteins

GST and GST-fusion proteins were purified as described previously [10]. For FLAG-tagged protein purification, 293T/17 cells were transfected with 10 mg of expression plasmids for DEAF1-FLAG or FLAG-Ku80 and wt-Ku70. Cell lysates were prepared in lysis buffer P (150 mM NaCl, 50 mM Tris pH 7.5, 1 mM EDTA, 1% Triton X100, 1 mM DTT, 1 mM NaF, and Complete Protease Inhibitor Cocktail (Roche)) on ice and cell debris was removed by centrifugation. Lysates were incubated with anti-FLAG beads overnight at 4°C. Proteins bound to the beads were washed four times with lysis buffer P and once with TBS (50 mM Tris pH 7.4, 150 mM NaCl) before elution with 200 ng/mL FLAG peptide in TBS for 30 min on ice.

### Isolation and Identification of DEAF1 Interacting Proteins

Human prostate PC-3 cells were cultured on 100 mm plates to subconfluency in DMEM/10% FBS. Cell lysates were prepared in lysis buffer P as above and incubated overnight at 4°C with either 5 mg of GST or 5 mg of GST-DEAF1 on GSH-Sepharose beads. The beads were washed six times with lysis buffer P, before the bound proteins were eluted with Laemmli sample buffer and separated on 10% SDS-PAGE gels. Proteins were visualized by Coomassie blue staining and unique bands were excised for protein identification by matrix-assisted laser desorption/ionization (MALDI) mass spectrometry at the W.M. Keck Foundation Biotechnology Resource Laboratory (Yale University).

### Immunoprecipitations and Immunoblotting

Immunoprecipitations (IP) experiments were performed as described [10]. Briefly, CV1 cells were cultured in DMEM/10% FBS on 100 mm plates and transfected with 5 mg of the indicated plasmids by calcium phosphate precipitation. Cell lysates were prepared in lysis buffer P as above and incubated with anti-FLAG beads overnight at 4°C. The beads were washed and proteins coimmunoprecipitating with the FLAG-tagged proteins were eluted with Laemmli sample buffer and analyzed by immunoblotting with the indicated antibodies as described [10].

### GST Pull-downs and DNA Effects on Protein-Protein Interactions

[^35^S]Methionine-labeled proteins were produced *in vitro* using the TNT Transcription/Translation System (Promega) and used in GST pull-down experiments as described previously [10] with the addition of either 15 mg of circular-closed plasmid DNA containing the DEAF1 promoter with multiple TTCG sequences or double-stranded oligos N52-69 ( TTCGGCTTCCCACTTCGG) or Mut 2 ( TT *TT*GCTTCCCACTT *TT*G) [9]. The double-stranded oligos were generated by PCR using overlapping primers, purified on 15% polyacrylamide gels, eluted out of gel slices overnight, ethanol-precipitated, resuspended in 50 mM KCl/10 mM Tris pH 7.5 and quantified before use.

### Electrophoretic Mobility Shift Assays

FLAG-DEAF1 and/or FLAG-Ku80/Ku70 complex proteins were purified from 293T/17 cells, incubated in binding buffer (100 mM KCl, 50 mM Tris 7.5, and 1 mM DTT) for 30 min at 25°C before the addition of ^32^P-labeled dsDNA probe, either N52-69 or Mut 2 and incubation for an additional 30 min. Complexes were separated on a 4% native polyacrylamide gel and visualized by PhosphorImager.

### DNA-PK Kinase Assay

One mg of either GST or GST-DEAF1 fusion proteins on GSH-Sepharose beads were incubated with DNA-PK protein (Promega) and [gamma-^32^P]-ATP in the absence or presence of 0.5 mg salmon sperm DNA for 30 min at 30°C. The beads were washed and the bound proteins were eluted with Laemmli sample buffer and separated by SDS-PAGE. Radiolabeled proteins were visualized with a PhosphorImager (Amersham Biosciences).

#### Localization of Fusion Proteins by Intrinsic Fluorescence and Immunofluorescence

CV-1 cells (150,000 cells/35 mm dish) were transfected with the indicated expression vectors by calcium phosphate precipitation. Twenty-four hours after transfection, cellular localization of GFP fusion and epitope-tagged DEAF1 and Ku70 proteins were determined as previously described [10].

## Results

### DEAF1 Interacts with Components of the DNA-PK Complex

**Figure 1:**
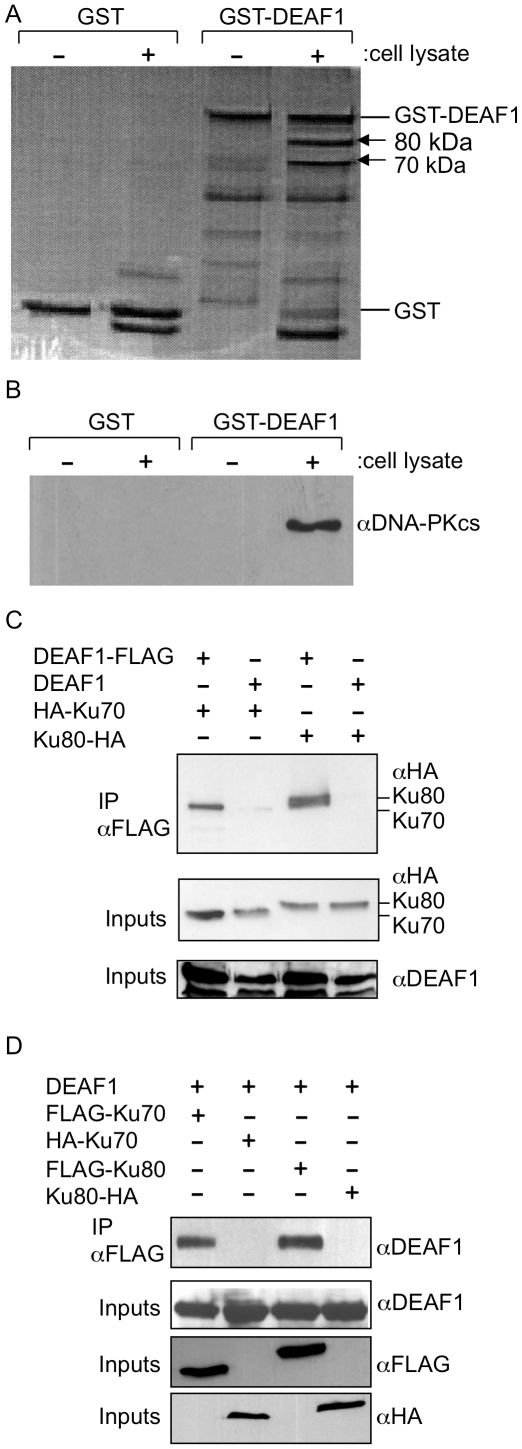
DEAF1 interacts with the DNA-PK complex. Recombinant GST and GST-DEAF1 fusion proteins were isolated from bacterial extracts, bound to glutathione-Sepharose beads, and incubated overnight in the presence (+) or absence (−, buffer only) of PC-3 cell lysates. Interacting proteins were eluted, separated by SDS-PAGE, and stained with Coomassie blue (**A**) or analyzed by Western blot with an antibody to DNA-PKcs (**B**). The 70 kDa and 80 kDa proteins (arrows) were determined by mass spectrometry to be Ku70 and Ku80. (**C**) CV1 cells were transfected with expression plasmids for DEAF1-FLAG and/or HA-tagged Ku proteins. Cell lysates were immunoprecipitated (IP) with anti-FLAG coupled beads followed by Western blot analysis with anti-HA antibody. (**D**) Reversing the epitopes described in (C), lysates of cells transfected with DEAF1 and FLAG-tagged or HA-tagged Ku proteins were immunoprecipitated with anti-FLAG coupled beads followed by Western blot analysis with anti-DEAF1 antibody. Levels of the proteins in 1.0% of the cell lysates (inputs) used in the immunoprecipitation reactions in C and D were assessed using the antibodies indicated. The results are representative of two independent experiments.

To identify proteins that interact with DEAF1, GST-DEAF1 pull-down experiments were performed using cell lysates from the PC-3 human prostate cell line. Two prominent protein bands of 70 kDa and 80 kDa were found unique to the GST-DEAF1/PC-3 cell lysate lane (Fig. 1A) and were subjected to MALDI mass spectrometry for identification of tryptic peptides. Peptide masses and matching peptides from a ProFound search are supplied in the Supplemental Data (File S1). For the 70 kDa protein, ProFound gave a score of 1.0 for Ku70 (gi|4503841) with 22% of the peptide masses matching and a Mascot search identifying the same protein. For the 80 kDa protein, a ProFound search gave a probability score of 1.0 for Ku80 (gi|108639450), with a Mascot search identifying the same protein, and 29% of the peptide masses matching. In analogous experiments, GST-DEAF1 pull-downs with human kidney 293T/17 and monkey kidney CV1 cell lysates produced similar banding profiles indicating that this interaction is not unique to PC-3 cells (data not shown). Ku70 and Ku80 are known to form a heterotrimeric complex with the catalytic subunit of DNA-PK (DNA-PKcs) and effect repair of double-stranded DNA breaks. To examine the potential interaction with this complex, GST-DEAF1 pull-downs were repeated and Western blot analysis was performed using an antibody against DNA-PKcs. A strong signal was observed for DNA-PKcs confirming that DEAF1 interacts with the DNA-PK complex (Fig. 1B).

To confirm the interactions of DEAF1 with Ku70 and Ku80 in cells, combinations of FLAG or HA epitope-tagged Ku70, Ku80, and DEAF1 constructs were transfected into CV1 cells and anti-FLAG coimmunoprecipitations were performed. DEAF1-FLAG was able to coimmunoprecipitate both HA-Ku70 and Ku80-HA (Fig. 1C), and reversing the epitopes showed that both FLAG-Ku70 and FLAG-Ku80 were able to coimmunoprecipitate DEAF1 (Fig. 1D).

### The SAND Domain of DEAF1 Interacts with the C-terminal End of Ku70

**Figure 2:**
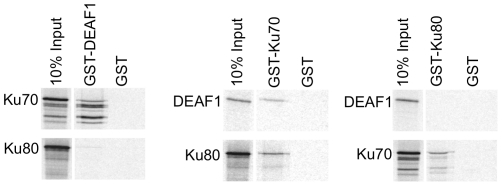
DEAF1 interacts with Ku70. GST pull-downs assays were performed by incubation of *in vitro* translated, [^35^S]methionine-labeled Ku70, Ku80, or DEAF1 with the indicated GST fusion proteins and GST. 10% of the *in vitro* translated proteins used in the pull-downs are shown on the left of each panel. The results are representative of two independent experiments.

Ku70 and Ku80 are relatively abundant proteins in human cell lines [13] that form a complex with each other, so we sought to determine if DEAF1 interacted directly with one or both proteins by *in vitro* pull-downs. GST fusion proteins were generated for DEAF1, Ku70, and Ku80 and used in GST pull-downs with *in vitro* translated proteins. GST-DEAF1 interacted with Ku70, but not Ku80 (Fig. 2, left panel). GST-Ku70 interacted with Ku80 as expected, and also interacted with DEAF1 (Fig. 2A, middle panel). GST-Ku80 interacted with Ku70, but not DEAF1 (Fig. 2, right panel). These results indicate that DEAF1 associates with the Ku/DNA-PK complex through direct interaction with the Ku70 subunit.

**Figure 3:**
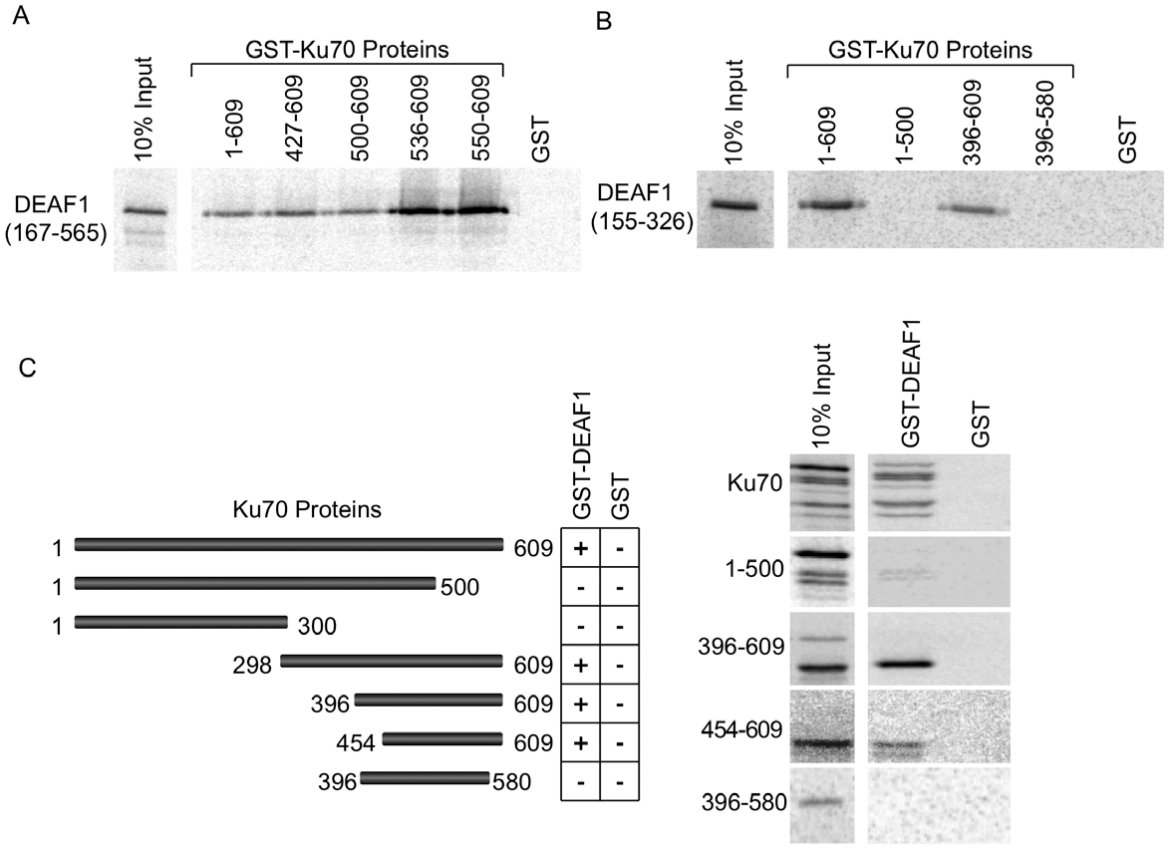
DEAF1 interacts with the C-terminal end of Ku70. (**A and B**) GST-Ku70 fusion proteins with N-terminal and/or C-terminal deletions of Ku70 were used in pull-downs with *in vitro* translated [^35^S]methionine-labeled DEAF1 (167–565) shown in (A) or DEAF1 (155–326) shown in (B). The results are representative of two independent experiments. (**C**) GST tags were reversed relative to (A) and (B) and GST-DEAF1 was used to pull-down *in vitro* translated Ku70 peptides (right panel). A schematic representation of all the Ku70 translated proteins tested is shown in the left panel. Results are summarized as a positive interaction (+) or no interaction (−).

The interaction domains of Ku70 and DEAF1 were further delineated by using various GST-Ku70 fusion proteins in pull-downs with two *in vitro* translated DEAF1 peptides: an amino-terminal (N-terminal) deletion of DEAF1 (amino acids 167–565) (Fig. 3A) and an internal peptide (amino acids 155–326) (Fig. 3B). Both of these peptides contain the DNA binding domain of DEAF1 (amino acids 167–306). The N-terminal deletion of DEAF1 interacted with all Ku70 N-terminal deletions that retained the carboxy-terminus (C-terminus) from amino acid 550–609 (Fig. 3A). The internal peptide of DEAF1 interacted with full-length Ku70 and the C-terminal protein of amino acids 396–609, but failed to interact with Ku70 proteins that lacked the C-terminus beyond amino acid 580, indicating that the C-terminal end of Ku70 is required for DEAF1 interaction (Fig. 3B). The experimental design was then reversed using GST-DEAF1 to pull-down *in vitro* translation products of Ku70 (Fig. 3C). These results confirmed that the C-terminal end of Ku70 beyond amino acid 580 is required to interact with DEAF1 (Fig. 3C). Thus, the smallest peptide of Ku70 shown to interact with DEAF1 was amino acids 550–609 (Fig. 3A), and this peptide contains the region (amino acids 578–583) that has been shown to bind and inhibit the proapoptotic protein Bax [14].

**Figure 4:**
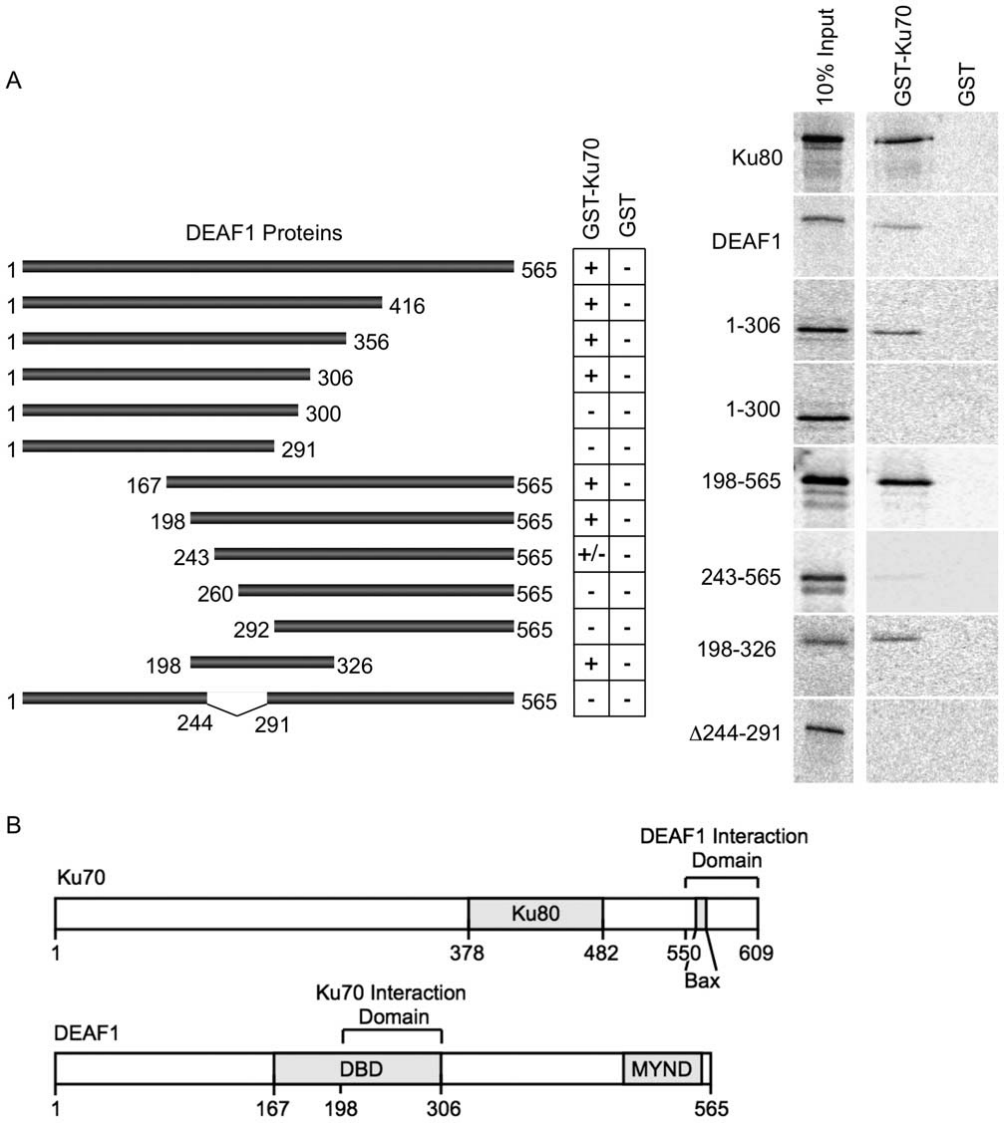
The DEAF1 DNA binding domain interacts with Ku70. (**A**) DEAF1 proteins with C-terminal and/or N-terminal deletions were radiolabeled with [^35^S]methionine by *in vitro* translation and used in GST pull-downs. 10% of the [^35^S]-labeled proteins (10% input) used in the pull-downs are shown to the left of the pull-down results obtained with GST-Ku70 and GST. Schematic representations of all the DEAF1 translated proteins tested are shown in the left panel. Results are summarized as a positive interaction (+) or no interaction (−). Ku80 interaction with GST-Ku70 was used as a positive control. (**B**) A schematic summary of the DEAF1 and Ku70 proteins and the interaction domains deduced from these experiments. Also shown are the Bax interaction domain [20], [24], the MYND (zinc binding) domain [25], and DBD (DNA binding domain).

The *in vitro* interactions of DEAF1 with Ku70 were then mapped with a more extensive set of deletions for DEAF1. DEAF1 interaction with Ku70 was lost upon deletion of 243 amino acids from the N-terminal end of DEAF1 and upon C-terminal truncation to amino acid 300 (Fig. 4A). The shortest DEAF1 peptide region to retain interaction with Ku70 contained amino acids 198–326, and the deduced interaction domain of amino acids 198–306 represents nearly all of the DEAF1 DNA binding domain. Schematics summarizing the interaction domains of Ku70 and DEAF1 are shown in Figure 4B.

### DEAF1-Ku70 Interaction does not Influence Cellular Localization

**Figure 5:**
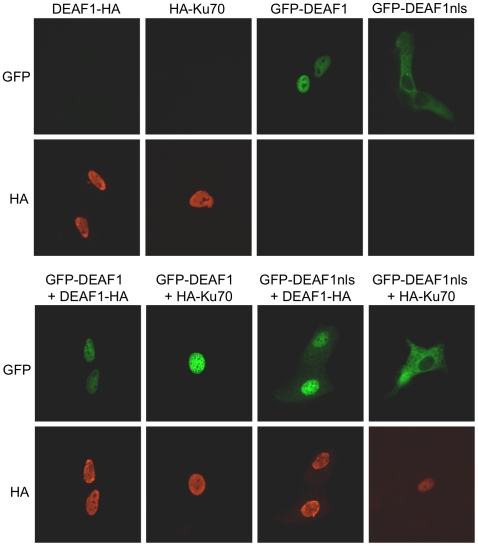
Ku70 is unable to relocalize a cytoplasmic form of DEAF1 to the nucleus. CV-1 cells were transfected either alone or in combinations with DEAF1-HA, HA-Ku70, GFP-DEAF1, or GFP-DEAF1nls (mutated nuclear localization signal). Cellular localization of HA-tagged proteins were determined by indirect immunofluorescence using anti-HA antibodies and secondary antibodies conjugated to CY3 and GFP-tagged proteins were determined by intrinsic GFP fluorescence.

*Wild-type* DEAF1, which localizes to the nucleus, has previously been shown to relocalize DEAF1 containing a mutation in the nuclear localization signal (nls) from the cytoplasm to the nucleus through DEAF1-DEAF1 protein interactions [10]. We sought to determine if the Ku70 interaction was sufficient to also cause the relocalization of the DEAF1 mutant. When expressed alone in CV-1 cells, DEAF1, Ku70, and GFP-DEAF1 all showed strong nuclear localization, while GFP-DEAF1nls showed strong cytoplasmic localization (Fig. 5). Co-expression of GFP-DEAF1 and Ku70 showed overlapping nuclear localization. When co-expressed with DEAF1, GFP-DEAF1nls relocalized to the nucleus, however co-expression of Ku70 and GFP-DEAF1nls produced no changes in either Ku70 nuclear localization or GFP-DEAF1nls cytoplasmic localization, indicating that the Ku70-DEAF1 interaction does not influence cellular localization of either protein.

### DEAF1 is Phosphorylated by DNA-PK *In Vitro*

**Figure 6:**
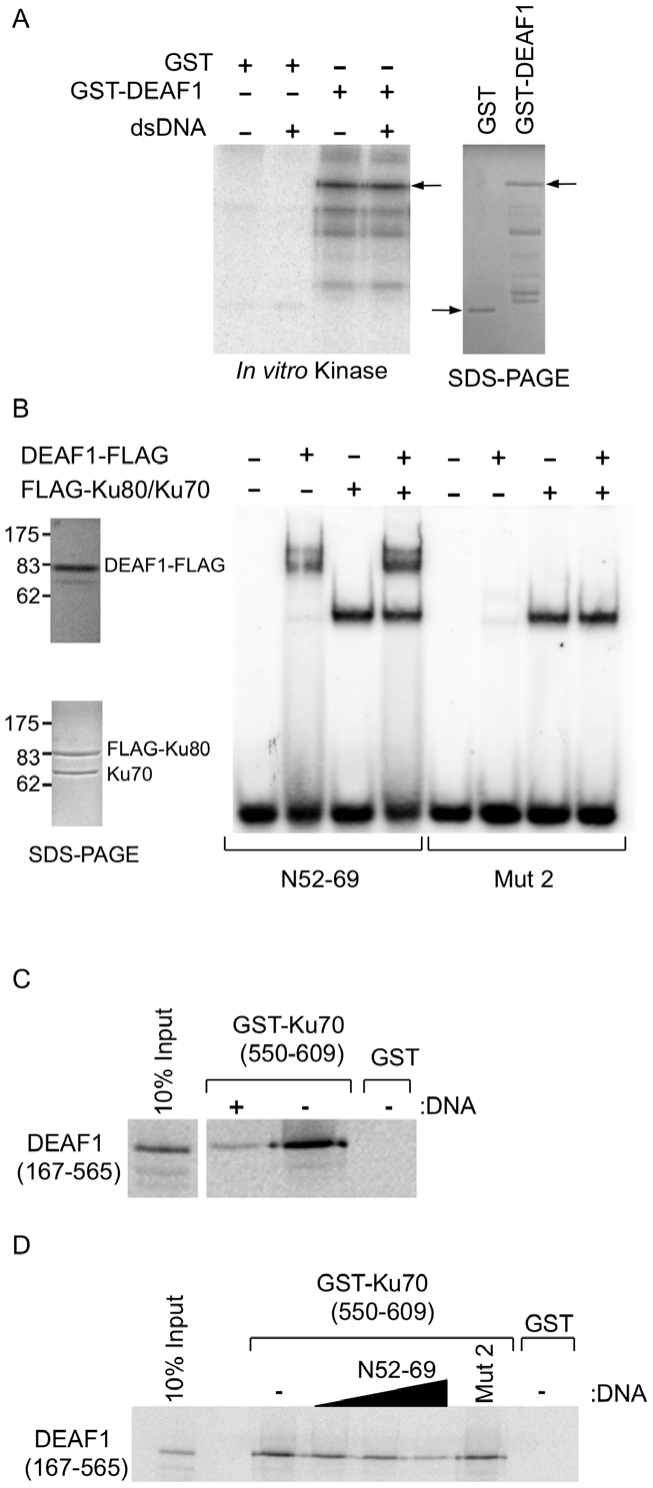
DNA-PK phosphorylates DEAF1, and sequence specific DNA competes with Ku70 for binding to DEAF1. (**A**) DNA-PK and [γ-^32^P]ATP were incubated with GST or GST-DEAF1 bound to glutathione-sepharose beads in the absence (−) or presence (+) of dsDNA (salmon sperm DNA sheared to ∼300 bp). Bound proteins were washed, eluted, and separated by SDS-PAGE; and the phosphorylated proteins were detected by autoradiography. Protein levels used in the reactions were assessed in separate SDS-PAGE gels stained with Coomassie blue. (**B**) Electrophoretic mobility shift assays were performed using the indicated FLAG proteins purified from HEK 293T/17 cells and either ^32^P-labeled dsDNA probes N52-69 (DNA ligand of DEAF1) or Mut 2 (mutated DNA ligand that DEAF1 does not bind). The purities of the proteins used in the assays are shown in Coomassie blue stained SDS-PAGE gels on the left. (**C and D**) GST pull-down experiments using GST-Ku70 (550–609) or GST with *in vitro* translated, [^35^S]methionine-labeled DEAF1 (167–565) were performed in the absence (−) or presence (+) of 15 µg of DEAF1 promoter DNA (*C*), or increasing amounts of N52-69 DNA (0.04 mg–0.6 mg) or 0.6 mg of Mut 2 DNA (*D*). 10% of the *in vitro* translated [^35^S]methionine-labeled proteins used in each pull-down experiment are shown on the left. The results are representative of two independent experiments.

DNA-PK is a serine/threonine protein kinase whose activity is increased by the presence of double-stranded DNA (dsDNA) ends and is responsible for the phosphorylation of a number of transcription factors and DNA binding proteins [15]. To investigate the possibility that DEAF1 is a substrate for DNA-PK, *in vitro* kinase assays were performed using DNA-PK with GST or GST-DEAF1 (Fig. 6A). DNA-PK phosphorylated GST-DEAF1 both in the absence and presence of dsDNA, indicating that DEAF1 is a substrate of DNA-PK and DNA is not necessary for the kinase activity. As a control, a similar amount of the GST protein, based on Coomassie blue staining of the proteins after SDS-PAGE, was used in the kinase reactions and GST was not phosphorylated by DNA-PK (Fig. 6A).

### Ku70 Does Not Interfere with DEAF1 Binding to DNA

Both the Ku complex and DEAF1 bind to DNA, therefore electrophoretic mobility shift assays were performed to determine if either protein would affect the DNA binding activity of the other. A double-stranded oligonucleotide called N52-69 has been previously shown to be a DNA binding target of DEAF1. It contains two TTCG motifs required for DEAF1 binding and binding is lost upon substitution of the two CG dinucleotides with two TT dinucleotides (Mut 2) [9], [10]. FLAG-epitope-tagged proteins were overexpressed and isolated from 293T/17 cells, and the purities of the proteins assessed by SDS-PAGE and Coomassie blue staining (Fig. 6B, left panels). Full-length DEAF1-FLAG isolated from mammalian cells produced a doublet of shifted bands with the N52-69 probe, but produced no shift with the Mut 2 probe (Fig. 6B, right panel). The observed doublet is not consistently observed and is possibly due to mammalian-specific protein modification of DEAF1 that does not occur when bacterially-expressed, shorter DEAF1 peptides are used [9], [10]. The Ku70/Ku80 complex bound both N52-69 and Mut 2 oligonucleotides, consistent with the Ku complex having a preference for free DNA ends rather than sequence specificity. Combination of the three proteins had no apparent affect on Ku or DEAF1 binding to N52-69 or Mut 2, indicating their interactions do not alter their respective DNA binding activity. In addition, there were no observed supershifts of the DNA probes, indicating that DEAF1, Ku, and DNA do not form a DNA-binding complex with an altered mobility (Fig. 6B).

The effects of DNA on the interactions between Ku70 and DEAF1 were also analyzed in GST pull-down experiments. We narrowed the study to the use of the C-terminal region of Ku70 (550–609), which binds DNA [16] and interacts with DEAF1 (Fig. 3A), and *in vitro* translated DEAF1 (amino acids 167–565). The addition of closed-circular plasmid DNA containing the DEAF1 promoter with multiple DEAF1 binding sites markedly decreased the interaction between DEAF1 and Ku70 (Fig. 6C). The addition of increasing amounts of double-stranded N52-69 oligonucleotide with a single DEAF1 binding site produced a modest decrease in the interaction between DEAF1 and Ku70 (Fig. 6D), while addition of Mut 2 oligo at levels equal to the maximal concentration of N52-69 oligo used, had no affect on DEAF1/Ku70 interaction. These results indicate that DNA with DEAF1 binding sites can displace or prevent Ku70 from binding to DEAF1 (Fig. 6C and 6D), while Ku70 is unable to displace DNA bound to DEAF1 (Fig. 6B).

## Discussion

DNA-dependent protein kinase is a heterotrimeric protein complex composed of three subunits: Ku70, Ku80, and the DNA-PK catalytic subunit (DNA-PKcs). DNA-PK has established functions in the non-homologous repair of DNA double strand breaks (NHEJ), immunoglobulin V(D)J gene rearrangement, and telomere maintenance (reviewed in [17]). DNA-PKcs may have functions independent of the holoenzyme and appears to interact directly with p53 to repress transcription of p21 and promote apoptosis [18]. Similarly, Ku70/Ku80 may function independently of DNA-PKcs by participating in an alternative pathway for the lengthening of telomeres [19]. Also, Ku70 has been reported to have an independent function from the DNA-PK holoenzyme, where it can function in the cytoplasm to bind and sequester Bax to reduce apoptosis [14], [15], [20].

In this study, we found that DEAF1 interacts with the DNA-PK complex through direct interaction with the Ku70 subunit. Deletion studies identified the interacting regions of the proteins as the centrally located DNA binding domain of DEAF1 and the C-terminal, Bax-interacting domain of Ku70 (Fig. 3–4). Both DEAF1 and Ku70/Ku80 can bind double-stranded DNA, however no supershifting of DNA was observed that would indicate formation of a complex among the three proteins and DNA (Fig. 6). DEAF1 binding to DNA was not affected by the addition of Ku70/Ku80, while DNA containing DEAF1 binding sites decreased DEAF1 interaction with Ku70. The EMSA results of Fig. 6 support the pulldown data of Fig. 4 and indicate that DEAF1 interacts with Ku70 through its DNA binding domain but this interaction is suppressed with an appropriate DNA ligand of DEAF1.

DEAF1 was *in vitro* phosphorylated by DNA-PK in a DNA independent manner (Fig. 6A). Similarly, DNA independent DNA-PK phosphorylation has been demonstrated for the thyroid hormone receptor-binding protein [21]. Thyroid hormone receptor-binding protein also interacts with DNA-PK through the Ku70 subunit and this may indicate that certain interactions with Ku70 eliminate the need for DNA to obtain DNA-PK kinase activity [21]. DNA-PK is able to phosphorylate substrate proteins at S/T-Q motifs [22]. Analysis of the DEAF1 primary amino acid sequence indicates potential DNA-PK phosphorylation sites at S43 and S48 (Scansite program) and T213 and T289 (NetPhosK program). T213 and T289 are both followed by a leucine residue such that these sites correspond to the S/T-hydrophobic amino acid motif demonstrated to be a target for DNA-PK phosphorylation [23]. Further studies are needed to determine the precise phosphorylation sites in DEAF1, whether DEAF1 is an *in vivo* substrate for DNA-PK, and if DNA-PK phosphorylation influences DEAF1 functions.

As expected, DEAF1 and Ku70 showed nuclear co-localization (Fig. 5). Previous studies had demonstrated that nuclear DEAF1 was able to relocalize DEAF1 with a NLS mutation from the cytoplasm to the nucleus [10]. Relocalization of mutant DEAF1 by nuclear DEAF1 was dependent on the second DEAF1-DEAF1 interaction domain that contains the DEAF1 nuclear export signal and has a potential coiled-coiled motif structure [10]. In contrast, nuclear Ku70 was unable to relocalize the cytoplasmic DEAF1 NLS mutant protein, suggesting that the Ku70-DEAF1 protein interaction is weaker than the self-interaction mediated by the coiled-coiled region of DEAF1.

The data presented in this study suggests that DEAF1 could be a target for phosphorylation, or modulate signal transduction of the DNA-PK complex through interaction with the Ku70 subunit. Alternatively, because DEAF1 binds at the C-terminal Bax binding domain of Ku70, DEAF1 might potentially affect apoptotic function when it is cytoplasmically localized and/or not bound to DNA. Future studies that examine the influence of DEAF1 on DNA-PK and Ku70 mediated apoptotic function appear warranted.

## Supporting Information

File S1 **ProFound search results using mass spectrometry peptide masses from the 70 & 80 kDa proteins that interact with DEAF1 and are identified as XRCC6 (Ku70) and XRCC5 (Ku80).**
(PDF)
