# Supplementary material for: Deformed Epidermal Autoregulatory Factor-1 (DEAF1) Interacts with the Ku70 Subunit of the DNA-Dependent Protein Kinase Complex
Source: PLoS One. 2012 Mar 19;7(3):e33404. doi: 10.1371/journal.pone.0033404 (PMC3307728; doi:10.1371/journal.pone.0033404)
Supplement: File S1 — ProFound search results using mass spectrometry peptide masses from the 70 & 80 kDa proteins that interact with DEAF1 and are identified as XRCC6 (Ku70) and XRCC5 (Ku80). (PDF) [file pone.0033404.s001.pdf]

Supplemental Data to “Deformed Epidermal Autoregulatory Factor-1 (DEAF1) Interacts with the Ku70 Subunit of the DNA-Dependent Protein Kinase Complex” by Philip J. Jensik, Jodi I. Huggenvik and Michael W. Collard

ProFound search results using mass spectrometry peptide masses from the 70 & 80 kDa proteins that interact with DEAF1 and are identified as Ku70 and Ku80.

ProFound - Search Result Details

Version 4.10.5
The Rockefeller University Edition

Details for rank 1 candidate in search BD96F7CA-02B8-7AC327BA

gi|4503841|ref|NP_001460.1| thyroid autoantigen 70kDa (Ku antigen); thyroid autoantigen 70kD (Ku antigen) [Homo sapiens]

gi|125729|sp|P12956|KU70_HUMAN ATP-dependent DNA helicase II, 70 kDa subunit (Lupus Ku autoantigen protein p70) (Ku70) (70 kDa subunit of Ku antigen) (Thyroid-lupus autoantigen) (TLAA) (CTC box binding factor 75 kDa subunit) (CTCBF) (CTC75)

gi|105163|pir||A30894 70K thyroid autoantigen - human

gi|15825664|pdb|1JEQ|A Chain A, Crystal Structure Of The Ku Heterodimer

gi|15825716|pdb|1JEY|A Chain A, Crystal Structure Of The Ku Heterodimer Bound To Dna

gi|178650|gb|AAA51733.1| p70 autoantigen

gi|250497|gb|AAB22381.1| Ku autoantigen p70 subunit [Homo sapiens]

gi|307095|gb|AAA36155.1| Ku protein subunit

gi|339667|gb|AAA61177.1| thyroid autoantigen

gi|5327042|emb|CAB46206.1| bK216E10.1 (thyroid autoantigen 70kD (Ku antigen)) [Homo sapiens]

gi|14249924|gb|AAH08343.1|AAH08343 thyroid autoantigen 70kD (Ku antigen) [Homo sapiens]

gi|14603133|gb|AAH10034.1|AAH10034 thyroid autoantigen 70kD (Ku antigen) [Homo sapiens]

gi|15082480|gb|AAH12154.1|AAH12154 Similar to thyroid autoantigen 70kD (Ku antigen) [Homo sapiens]

gi|17390603|gb|AAH18259.1|AAH18259 thyroid autoantigen 70kD (Ku antigen) [Homo sapiens]

```
Sample ID :  RD65, Jensik [Pass:0]
Measured peptides      : 27
Matched peptides       : 11
Min. sequence coverage: 22%
```

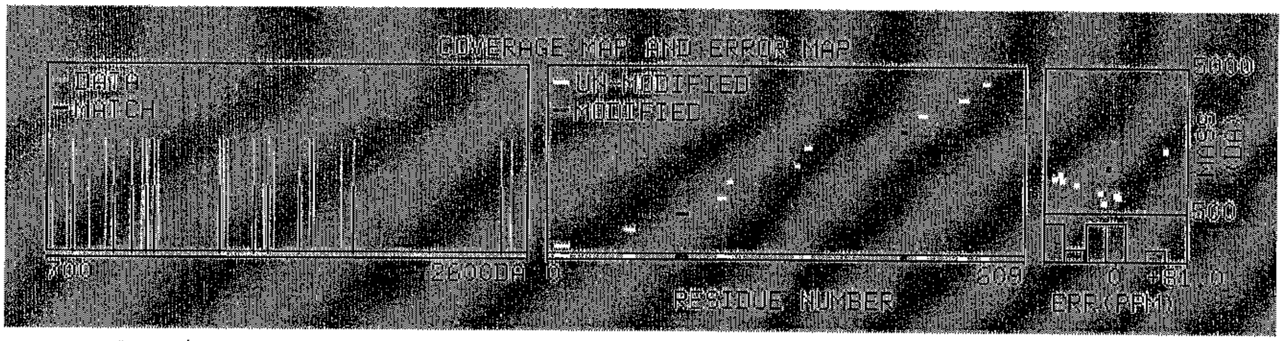

Measured Mass (M)	Avg/ Mono	Computed Mass	Error (ppm)	Residues Start	To	Missed Cut	Peptide sequence
809.393	M	809.403	-12	319	325	0	SQIYGSR
961.454	M	961.450	4	231	238	0	VHFEESSK
1032.508	M	1032.508	0	332	339	1	EETEELKR
1088.539	M	1088.553	-13	452	461	0	IMATPEQVGK (1)+O@M;
1124.505	M	1124.523	-16	557	565	0	VEYSEEELK
1387.623	M	1387.682	-43	219	230	0	DIISIAEDEDLR
1519.628	M	1519.719	-60	527	539	0	ELVYPPDYNPEGK
1572.707	M	1572.814	-68	101	114	0	NIYVLQELDNPGAK
1702.700	M	1702.806	-62	475	488	0	SDSFENPVLQQHFR
1917.838	M	1917.852	-7	166	182	0	IMLFTNEDNPHGNDSAK (1)+O@M;
2500.129	M	2499.987	57	10	31	0	TEGDEEAEEEQEENLEASGDYK

ProFound - Search Result Details

Version 4.10.5
The Rockefeller University Edition

Details for rank 1 candidate in search BB62F265-04EC-788F2586

gi|10863945|ref|NP_066964.1| ATP-dependant DNA helicase II; X-ray repair complementing defective repair in Chinese hamster cells 5 (double-strand-break rejoining; Ku autoantigen, 80kD); X-ray repair, complementing defective, repair in Chinese hamster; DNA repair protein XRCC5 [Homo sapiens]

gi|125731|sp|P13010|KU86_HUMAN ATP-dependent DNA helicase II, 80 kDa subunit (Lupus Ku autoantigen protein p86) (Ku86) (Ku80) (86 kDa subunit of Ku antigen) (Thyroid-lupus autoantigen) (TLAA) (CTC box binding factor 85 kDa subunit) (CTCBF) (CTC85) (Nuclear factor IV) (DNA-repair protein XRCC5)

gi|88066|pir||A32626 Ku antigen 80K chain - human

gi|307093|gb|AAA59475.1| Ku antigen

gi|307094|gb|AAA36154.1| Ku (p70/p80) subunit

gi|17512093|gb|AAH19027.1|AAH19027 X-ray repair complementing defective repair in Chinese hamster cells 5 (double-strand-break rejoining; Ku autoantigen, 80kD) [Homo sapiens]

```
Sample ID :   RD76  [Pass:0]
Measured peptides      : 47
Matched peptides       : 21
Min. sequence coverage: 29%
```

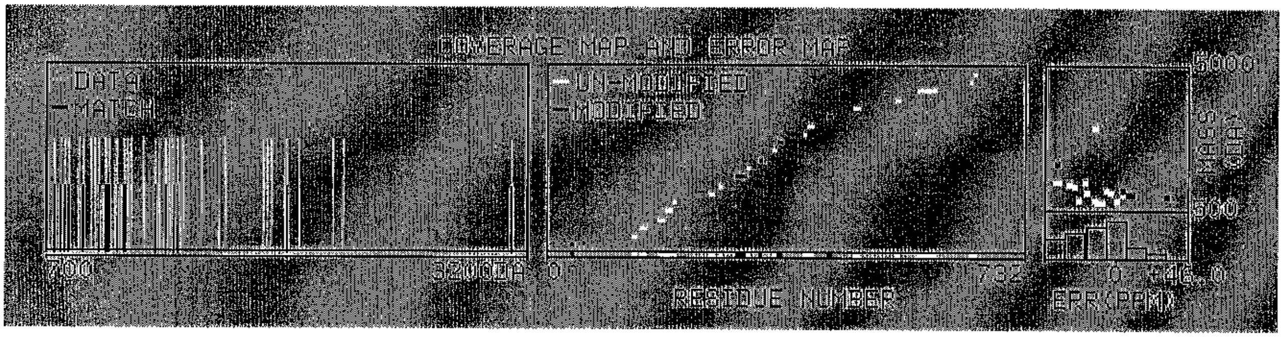

Measured Mass (M)	Avg/ Mono	Computed Mass	Error (ppm)	Residues Start	To	Missed Cut	Peptide sequence
703.352	M	703.361	-13	348	353	0	SSQVQR
744.406	M	744.413	-9	266	271	0	SILQER
781.401	M	781.412	-14	655	660	0	FNNFLK
794.356	M	794.355	1	649	654	0	FSEEQR
802.399	M	802.418	-23	196	202	0	GITEQQK
814.397	M	814.397	0	395	400	1	YAYDKR
893.408	M	893.379	32	326	332	0	VDEEQMK<br>(1)+O@M;
976.501	M	976.497	4	308	315	0	EDIIQGFR
1008.549	M	1008.542	7	37	44	0	VITMFVQR<br>(1)+O@M;
1018.490	M	1018.479	11	432	439	0	QYMFSSLK<br>(1)+O@M;
1030.603	M	1030.606	-3	535	543	0	TLFPLIEAK
1072.603	M	1072.623	-19	251	260	0	LTIGSNLSIR
1098.557	M	1098.553	4	355	363	0	FFMGNQVLK<br>(1)+O@M;
1108.596	M	1108.602	-6	185	195	0	LGGHGPSFPLK
1265.700	M	1265.734	-26	145	155	0	SQLDIIIHSLK
1316.632	M	1316.672	-30	131	141	0	HIEIFTDLSSR
1363.525	M	1363.575	-36	172	184	1	EDGSGDRGDGPFR
1376.702	M	1376.756	-39	401	413	0	ANPQVGVAFPHIK
1379.653	M	1379.681	-20	470	481	0	TDTLEDLFPTTK
1955.791	M	1955.866	-38	292	307	0	ETVYCLNDDDETEVLK<br>(1)+C3H5ON@C;
